# Remote mentoring in laparotomic and laparoscopic cancer surgery during Covid-19 pandemic: an experimental setup based on mixed reality

**DOI:** 10.1080/10872981.2021.1996923

**Published:** 2021-10-29

**Authors:** Michele Simone, Rocco Galati, Graziana Barile, Emanuele Grasso, Raffaele De Luca, Carmine Cartanese, Rocco Lomonaco, Eustachio Ruggieri, Anna Albano, Antonello Rucci, Giuseppe Grassi

**Affiliations:** a Department of Oncology Surgery, IRCCS Istituto Tumori “Giovanni Paolo II”, Bari, Italy; b Dipartimento Ingegneria Innovazione, Università del Salento, Lecce, Italy

**Keywords:** Virtual reality, mixed reality, laparotomic surgery, smartglasses, laparoscopic surgery

## Abstract

In this paper, Mixed Reality (MR) has been exploited in the operating rooms to perform laparoscopic and open surgery with the aim of providing remote mentoring to the medical doctors under training during the Covid-19 pandemic. The employed architecture, which has put together MR smartglasses, a Digital Imaging Player, and a Mixed Reality Toolkit, has been used for cancer surgery at the IRCCS Hospital ‘Giovanni Paolo II’ in southern Italy. The feasibility of using the conceived platform for real-time remote mentoring has been assessed on the basis of surveys distributed to the trainees after each surgery.

## Introduction

The COVID-19 crisis has greatly impacted almost every person’s life on earth, and governments around the world have taken extraordinary measures to reduce the spread of the virus. Italy has been one of the most seriously affected countries in the world [1]. Educating the next generation of physicians and surgeons is still necessary, even during a pandemic; technological advances may be leveraged to facilitate remote training of surgeons while still allowing for real-time feedback. Mixed Reality (MR) represents a tool where the merging of real and virtual worlds generates an environment characterized by the real-time coexistence of physical and digital objects [2–6].

By exploiting our expertise in smartglasses-based open abdomen surgeries [7], we have used MR in the operating rooms for remote mentoring of the medical staff under training during Covid-19 pandemic. The study evaluates the implementation of MR in the cancer surgeries carried out at the IRCCS Hospital ‘Giovanni Paolo II’ in the city of Bari (Italy). The feasibility of introducing the MR smartglasses during surgery for real-time remote mentoring has been assessed via feedback and surveys provided by the medical staff under training.

## Substance of the innovation

**Figure 1.**
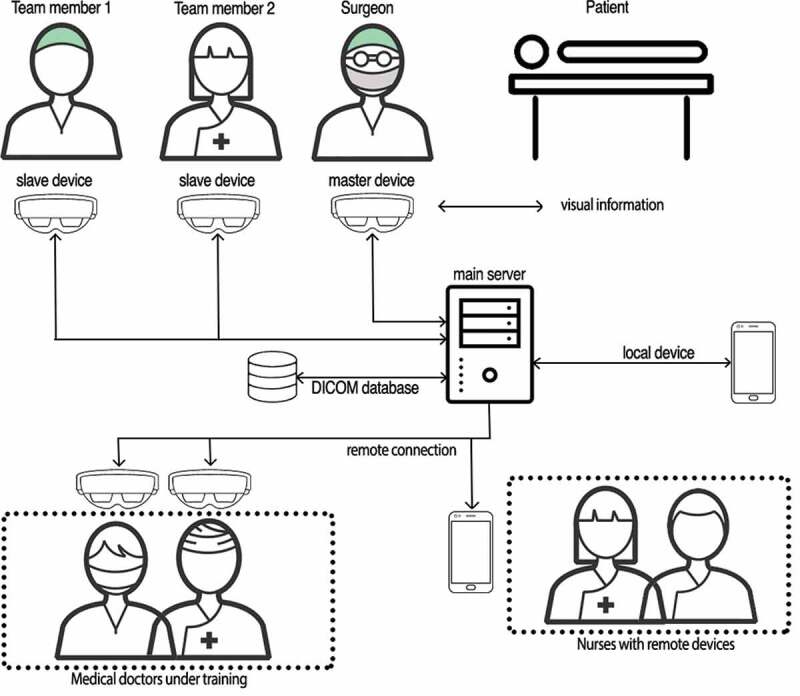
The adopted architecture for remote mentoring includes MR smartglasses, a digital imaging player, a DICOM database containing the medical images and a mixed reality toolkit

A new advanced experimental setup based on the use of MR smartglasses [8] has been employed at the IRCCS Hospital from March 2020 to December 2020, with the aim to carry out remote mentoring when face-to-face training activities were suspended by law due to the Covid-19 pandemic (Figure 1).

The members of the surgical team have been equipped with the MR headset. They can visualize the results of medical screenings (i.e., radiography, magnetic resonance imaging, blood tests, etc.) and can add visual information on the patient’s body using MR tools. These data generated by the smartglasses are shared with the medical doctors under training via their laptops and Android-based smartphones. The conceived architecture includes several tools for aligning virtual objects with the physical world in both the smartglasses and the mobile devices. Note that the considered smartglasses, while sending scene information to the mobile device, update its local application content, so that all the team’s members share the surgeon’s point of view [9]. This enables the training of medical doctors to be effective, because they can observe the real scene with all the augmented information placed by the surgeon, including virtual tooltips on the patient’s body to highlight regions of interest.

## Addressed educational problem

In most of the Italian hospitals, the training activity was suspended due to the Covid-19 pandemic. At the IRCCS Hospital, this educational problem has been overcome by virtue of the innovation described in Section 2. We selected 12 general oncological surgeries performed using laparotomy or laparoscopic techniques. All patients were subject to preoperative CT, MRI, or PET scan for accurately defining the extension of the neoplasm in nearby organs. The day before each surgery, a computer engineer gave a 2-hour seminar to each member of the surgery team and to the trainees, with the aim to practically show how to exploit all the capabilities offered by the platform. The two most complicated surgeries are now described by highlighting the addressed educational issues.

### Right adrenal carcinoma infiltrating the vena cava

**Figure 2.**
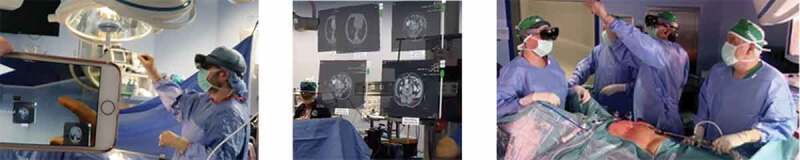
The surgeon while resizing a virtual image (left); the virtual images placed on the real scene (center); the surgeon while dragging a digital photo (right)

The surgeons wearing the MR smartglasses performed the surgery of a right adrenal carcinoma infiltrating the vena cava with the possibility of simultaneously viewing TC or MR images of the patient in the form of holograms. The surgeons also explored the virtual content positioned in different points of the space with a simple gesture of the hand (Figure 2).

The support of MR smartglasses has enabled the trainees to analyze the rich vascularization of the tumor mass and its contiguity with the vena cava, besides assuring better identification of anatomical variants and lymph nodes [10]. Note that, without the proposed innovation, it would be impossible for the trainees to benefit of these visualizations, because face-to-face training was suspended.

### Gastrectomy

**Figure 3.**
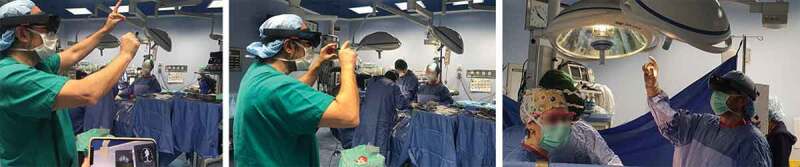
The surgeon performs a ‘drag and drop’ gesture during a gastrectomy to rotate and scale a virtual image

The surgeons wearing the smartglasses carried out a gastrectomy while performing rotation and scaling with simple ‘drag and drop’ gestures (Figure 3). By virtue of MR tools, the surgeons were able to add virtual objects (i.e., images, texts, and videos) on the patient body, using them as guidance or notes during the interventions.

The smartglasses enabled the trainees to correctly identify the anatomical variations of the gastric vascular vessels [11]. Specifically, by following the instructions of the surgeons in the operating room and by consulting the radiological images via the smartglasses, the trainees have identified an anatomical variant for the right gastric artery that originated from the gastroduodenal artery. Without the proposed innovation, it would be impossible for the trainees to benefit of this medical knowledge.

## Outcomes of training

Referring to the carcinoma, a 10-points Likert-type scale survey (Table 1) has been used to evaluate the experience of the five trainees (‘1’ represents ‘negative experience’, ‘10’ indicates ‘top satisfaction’).

**Table 1.** Feedback from the five medical doctors under training: right adrenal carcinoma. For each reported item, the total score represents the average over the five trainees

Evaluations on smartglasses used for telementoring activities	
Items	Total score
Q1: How would you rate the real time-interaction during the surgery?	8
Q2: Did the smartglasses help you to focus on the activities?	9
Q3: Did the smartglasses enhance your degree of attention?	9
Q4: How would you rate the instant feedback you received?	8
Q5: How helpful was the platform with reference to the acquired skills?	8
Q6: How would you rate your overall experience?	8

Table 1 regards one laparotomic surgery (performed on 7 April 2020 at the IRCCS Hospital in Bari) to remove a right adrenal carcinoma infiltrating the vena cava. Table 1 reports the items contained in the surveys, which were distributed to the five trainees immediately after the surgery.

Referring to the gastrectomy (Table 2), the results highlight an overall satisfaction, because the use of MR generates an immersive environment, which gives a first-hand experience by increasing the focus on the training activities (see ‘overall experience’). In particular, identifying an anatomical variant for the right gastric artery has been very useful for young surgeons from the perspective of remote mentoring (see ‘acquired skills’), if we consider that, in most of the hospitals, the training activity was suspended due to the pandemic. Note that the gastrectomy was performed using laparoscopic surgery on 12 April 2020 at the IRCCS Hospital. Finally, a survey regarding all the 12 surgeries was given to the five trainees immediately after the twelfth surgery (Table 3).

**Table 2.** Feedback from the five medical doctors under training: gastrectomy

Evaluations on smartglasses used for telementoring activities	
Items	Total score
Q1: How would you rate the real time-interaction during the surgery?	7
Q2: Did the smartglasses help you to focus on the activities?	9
Q3: Did the smartglasses enhance your degree of attention?	9
Q4: How would you rate the instant feedback you received?	9
Q5: How helpful was the platform with reference to the acquired skills?	9
Q6: How would you rate your overall experience?	9

**Table 3.** Survey from the telementoring course attendees regarding all the 12 surgeries (scale from 1 to 10, low to high)

Final feedback for telementoring activities	
Items	Total score
Q1: How useful were the smartglasses for the overall training?	8
Q2: How would you rate the quality of the training course?	7
Q3: How useful was the virtual content delivered via the platform?	7
Q4: How would you rate the shared multimedia content?	8
Q5: How would you rate the usefulness of this course to your work?	8
Q6: How would you rate the safety (during the training) with reference to Covid-19?	7

## Lesson learned

Besides the advantages of the approach (i.e., enabling an otherwise impossible training activity during the lockdown as well as real-time sharing multimedia contents such as blood tests and medical imaging), we have also encountered some difficulties, which have mainly regarded the complex technical tuning of the hardware and software platform (i.e., synchronizing the behaviours of the smartglasses with the Digital Imaging Player and the DICOM database containing the medical images). Once we had the working platform, it has been used at the IRCCS Hospital also when the Covid-19 restrictions have been lifted. We believe that the tool could be adopted by other institutions in the future during other potential pandemics, also by virtue of the low-cost employed equipment.
